# Unexplained neurological events during bathing in young people: Possible association with the use of gas geysers

**DOI:** 10.4103/0972-2327.41877

**Published:** 2008

**Authors:** Prabhjeet Singh, Anuraag Lamba, Rajinder Bansal, Gagandeep Singh

**Affiliations:** Department of Neurology, Dayanand Medical College, Ludhiana - 141 001, Punjab, India

**Keywords:** Liquefied petroleum gas, carbon monoxide, intoxication

## Abstract

Here, we report sudden, unexplained neurological collapse in 14 young people while bathing with hot water associated with the use of liquefied petroleum gas (LPG)-based water heaters (gas geysers) in ill-ventilated bathrooms. None of the patients reported any circumstantial evidence of seizures or prior epilepsy. One patient developed cortical blindness and demonstrated posterior leucoencephalopathy on imaging studies. The remaining patients made rapid and excellent recovery without any residual neurological sequelae. In these cases, the results of all routine investigations, i.e., serum chemistry, brain imaging (computed tomography in 2 and magnetic resonance imaging in 10) and electroencephalography were normal. The clinical clustering of these cases in winter months with similar presentations of reversible encephalopathy probably indicates an inhalational toxin exposure. Therefore, we postulate a hypothesis that harmful emissions consisting of carbon monoxide (CO), hydrocarbon gases (HC) and nitrogen oxides (NOx), produced by incomplete combustion of LPG might be responsible for the cellular injury and subsequent transient neurological deficits. Physicians should be aware of this entity in order to avoid misdiagnosis of this condition as seizures, and a public awareness should also be created regarding the proper use of these devices.

## Introduction

We report the occurrence of unwitnessed, unexplained neurological events involving mostly of a sudden and reversible loss of consciousness and collapse in young people taking prolonged, traditional Indian hot water baths using a bucket and mug in small and ill-ventilated bathrooms. Although the sudden loss of consciousness in the bathroom can be due to a number of causes ranging from seizure, head injury, stroke involving subarachnoid hemorrhage, cardiac events, syncopal episodes to various poisonings and toxin exposures, we hereby attempt to draw the attention of the readers to a new possible cause-carbon monoxide poisoning. In the era of angithees, sleeping in closed bedrooms was a known cause of loss of consciousness. This cause has resurfaced with another source-liquefied petroleum gas (LPG)-based water heaters, also known as gas geysers, are becoming increasingly popular as a method to heat water during the winters owing to lower installation and running costs and the practicability of their use during periods of erratic electrical power supply. The loss of consciousness can probably be associated with the exposure to toxic gases, predominantly carbon monoxide, released during the combustion of LPG in these gas geysers.

## Case Reports

Fourteen cases, the prototype of which is described below (case 1) were noted in the outpatient clinic and two patients (case 2) were identified from the inpatient records of Dayanand Medical College Hospital, Ludhiana. This hospital serves the urban and rural ethnic populations of the states of Punjab, Himachal Pradesh and Rajasthan in North-West India. The temperature here is raw with both extreme summers and winters, imposing the use of hot water for bathing in the cold weather.

### Case 1

A 16-year-old boy collapsed while bathing with hot water on a Sunday morning. When he did not emerge from the bathroom for nearly 45 min, his family members became suspicious and broke open the door. He was found unconscious and rushed to medical aid where he gradually regained consciousness over the next one hour. The witnesses did not report any changes in the skin color, abnormal posturing or clonic movements. At the medical facility, his vital signs were reported to be normal. He was discharged from the medical facility within few hours with a prescription of carbamazepine. The next day, the boy presented to the outpatient clinic. He reported a feeling of dizziness after approximately 25 min in a hot water bath but no definite aura. He did not notice any tongue bite or a feeling of soreness in muscles to indicate the occurrence of a convulsion. He was also unable to recount the occurrence of a prior seizure-precipitating event such as sleep deprivation or drug intoxication or previous seizures or myoclonic jerks or a history of seizures in the family. The hot water used for his bath came through a gas geyser that was located completely (with burner and cylinder) inside the small sized bathroom (3 × 4'). Although there was a small ventilator in the bathroom, it was shut due to cold weather.

The results of complete physical, neurological and cardiac examinations were unremarkable. The results of a two-hour awake and sleep electroencephalogram, noncontrast magnetic resonance imaging (MRI) of the brain, electrocardiogram and serum chemistries, blood gas analysis and hemogram were normal. A diagnosis of neurological collapse due to possible carbon monoxide intoxication was offered and no specific treatment was administered. One year later, the boy remains asymptomatic.

### Case 2

A 14-year-old girl was found unconscious in the bathroom while bathing with hot water heated in a gas geyser installed within the room. She was brought to the Emergency Room in an unconscious state. Upon physical examination, the heart rate was 120 beats/m; respiratory rate, 20/m and blood pressure, 130/70 mmHg. The results of general and cardiac examinations were normal. The girl remained unconscious in response to deep painful stimuli. Pupils were 3 mm, equal in size and showed normal reaction to light. She was able to move all the four limbs in response to deep painful stimuli. All deep tendon reflexes were normally elicitable and both plantar responses were extensor. Examination of the ocular fundus did not reveal any abnormality. There were no signs of meningeal irritation.

**Figure 1:**
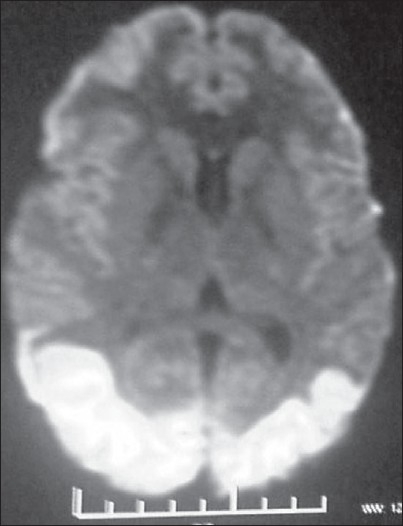
Diffusion-weighted MRI of the brain of the second patient showing hyperintensities in the bilateral occipital lobe

The results of routine hemogram and serum chemistry were normal. Electrocardiogram revealed sinus tachycardia. The girl regained consciousness over the next 3 h, although she remained confused for the next 24 h. Few days later when her consciousness was clear, she complained of diminution of vision in both eyes along with intermittent headaches. Bedside visual examination revealed only perception of light, thus precluding visual field assessment. Both pupils reacted well to light. The results of ocular and fundus examinations were unremarkable. Routine MRI of the brain was essentially normal; however, diffusion-weighted imaging revealed bilateral hyperintensities in the occipital lobes [Figure 1]. MR angiography revealed normal cerebral vascular anatomy, and the result of cardiac echo examination was normal. Visual evoked response testing revealed the absence of any waveform at the occipital electrodes. The blood pressure remained normal throughout the one week stay at the hospital. Later, the patient was shifted to another hospital and inquires made from this facility revealed that her vision improved with no specific treatment over a period of two to three weeks. A provisional diagnosis of toxic encephalopathy due to LPG combustion products was made.

### Other cases

The remaining 12 patients presented for the evaluation of neurological collapse, all of which occurred in ill-ventilated bathrooms, while taking a prolonged bath with hot water drawn through a gas geyser fitted within the room. All patients recovered consciousness within few minutes to hours. There were no residual neurological sequelae. None reported circumstantial evidence of seizures or prior occurrence of epilepsy. The findings of serum chemistry, brain imaging (computed tomography in 2 and MRI in 10) and electroencephalography (EEG) were normal. Psychometric evaluations performed in two cases were unremarkable.

## Discussion

The cases described herein were common, such as the occurrence of sudden, unexplained and unwitnessed neurological collapse in young people bathing with hot water. Besides, in all instances, it is noteworthy that the water used for bathing was heated by gas geysers fitted within ill-ventilated and small bathrooms, and there was a clustering of such cases in winter months. Finally, all except one patient made excellent and rapid neurological recovery and a follow-up revealed normal imaging and EEG. Although it is possible that these patients did experience seizures, there is no circumstantial or other supportive evidence (e.g., prior seizures or abnormal EEG or imaging) to support this contention in any of the patients. However, the evidence, although not conclusive, suggests that the neurological episodes were related to the operation of gas geysers. Hence, we postulate that harmful emissions from the combustion of LPG were responsible for the neurological collapse.

Gas geysers are being increasingly employed for domestic heating of water in the absence of a centralized source of hot water in several developing countries. Regulatory agencies have developed guidelines for the use of these geysers, including the use of timers, site of installations and dimensions and ventilation requirements of the room in which they are fitted. LPG is used to heat water in these gas geysers. An exception to these recommendations, however, was that all patients in this report used gas geysers, which were fitted in small and ill-ventilated bathrooms.

Harmful emissions from combustion of LPG include carbon monoxide (CO), hydrocarbon gases (HC) and nitrogen oxides (NOx). Carbon monoxide is generated in the exhaust as a result of incomplete combustion of fuel. It is a toxic, colorless and odorless gas that can accumulate rapidly and reach concentrations, which are dangerous for humans. It quickly binds to hemoglobin with an affinity 200–250 times greater than that of oxygen to form COHb. The resulting decrease in the arterial oxygen content and shift of the oxyhemoglobin dissociation curve to the left explain the acute hypoxic symptoms (primarily neurologic and cardiac) noted in patients with carbon monoxide poisoning. Nitrogen oxides (including nitric oxide and nitrogen dioxide) are generated from nitrogen and oxygen under the high temperature and pressure conditions during LPG combustion, which is also potentially toxic.

Fortunately, all patients, except one, made excellent and rapid neurological recovery. This is possibly due to prompt intervention in the form of removal from the bathroom by family members.[1,2] The neurological sequelae in a single patient described above are of interest since similar clinical and MRI abnormalities have not been reported before. The clinical and imaging findings (posterior white-matter hyperintensities) in the absence of any angiographic or cardiac abnormality upon investigations and the clinical reversibility over weeks suggest the possibility of posterior leucoencephalopathy. No precipitating cause for the leucoencephalopathy was noted since the patient's blood pressure remained normal throughout the hospital stay. We suggest that carbon monoxide or possibly another LPG exhaust component may be responsible for the posterior leucoencephalopathy in the absence of any other precipitating cause. A recently developed hypothesis suggests that carbon monoxide poisoning is followed by reoxygenation injury to the central nervous system. Hyperoxygenation facilitates the production of partially reduced oxygen species, which in turn can oxidize essential proteins and nucleic acids, resulting in typical reperfusion injury.[2] In addition, carbon monoxide exposure has been shown to cause lipid peroxygenation (degradation of unsaturated fatty acids), leading to reversible demyelinization of central nervous system lipids.[3–5]

## Conclusion

We attempt to draw the attention of physicians to the entity of LPG fume poisoning as a cause of unexplained neurological collapse in bathrooms. There is also a need to create public awareness regarding the adherence to guidelines for the installation of and provision of ventilatory safeguards while using gas geysers.
